# A meta-analysis-guided sampling plan for estimating the prevalence of 10 zoonotic and 4 transboundary animal diseases across India

**DOI:** 10.3389/fvets.2026.1919244

**Published:** 2026-10-16

**Authors:** K. P. Suresh, M. Ashwini, P. Azhahianambi, Subeer S. Majumdar, Nagendra R. Hegde

**Affiliations:** 1 Division of Statistics, Indian Council for Agricultural Research – National Institute of Veterinary Epidemiology and Disease Informatics, Bengaluru, India; 2 Translational Research Platform for Veterinary Biologicals, Centre for Animal Health Studies, Tamil Nadu Veterinary and Animal Sciences University, Chennai, India; 3 Biotechnology Research Innovation Council – National Institute of Biotechnology, Hyderabad, India

**Keywords:** meta-analysis, One Health, prevalence, surveillance, systematic review, transboundary animal disease, zoonotic disease

## Abstract

**Background and Objectives:**

Both natural and anthropogenic factors impact the emergence, sustenance, and spread of zoonotic diseases and Transboundary Animal Diseases (TADs). Surveillance of such diseases depends on establishing baseline prevalence data, which necessitates a scientifically rigorous plan for data collection. This study aimed to establish a robust plan for estimating the prevalence of 10 zoonotic diseases (brucellosis, tuberculosis, Q-fever, scrub typhus, Japanese encephalitis, Crimean-Congo haemorrhagic fever, cysticercosis, cryptosporidiosis, listeriosis, salmonellosis) and four TADs (swine influenza, porcine reproductive and respiratory disease, lumpy skin disease, African swine fever) in India.

**Methods:**

Based on a systematic review and meta-analysis conducted in accordance with PRISMA Guidelines, using random effects model with heterogeneity quantified by the I^2^ statistics estimate of preliminary prevalence rates, and incorporating predetermined sensitivity and specificity values for the assays to be employed, a two-stage stratified random surveillance plan was generated with 95% confidence level, using an in-house developed epi-calculator, with the village as the stratification variable for the targeted animal species in different states of India.

**Results:**

The pooled prevalence estimates of the 14 diseases ranged from 4 to 30% (95% confidence intervals reported in the corresponding meta-analysis estimates). Based on these pooled prevalence estimates, a village-level surveillance plan comprising 34,975 samples across 2,018 villages was generated for India.

**Conclusion:**

This is the first scientifically rigorous meta-analysis-based sampling plan under one platform for many diseases. This could facilitate a robust prevalence study, which could serve as an effective tool for monitoring zoonotic diseases and TADs that are a threat to India.

## 1 Introduction

One Health approach is a holistic, cross-disciplinary approach that operates at various levels, addressing health at the intersection of humans, animals, and the environment. It involves collaboration across sectors and is essential for managing diseases. The approach is especially important for epidemics and zoonotic diseases, which have a significant burden and disproportionately impact poorer communities, hindering poverty reduction (1–3). India’s diverse geographical and socioeconomic landscape not only demands but also highlights the need for implementing strategies for effective coordinated action to achieve One Health (2, 4).

India has the world’s highest livestock population and the second-largest human population. Amid shrinking green cover and wildlife habitats on one side and the increased need for food security on the other, the country faces increased interaction among humans, livestock, and wildlife. To address resulting issues such as outbreaks and risks of zoonotic diseases and antimicrobial resistance, the government has seriously considered the implementation of the One Health approach. Among the several measures was the support in 2021 by the Department of Biotechnology, Ministry of Science & Technology, to set up a consortium of 28 centres aimed at connecting the veterinary, wildlife and medical sectors. This initiative focuses on nationwide studies to establish baseline prevalence data for significant zoonotic diseases and certain transboundary animal diseases (TADs) that have recently affected northeast India. This represents the first and the largest One Health-related initiative in India in the post-COVID era (5).

An interdisciplinary network is essential for an effective One Health-based approach to disease surveillance. Timely epidemic detection is crucial to prevent spread, reduce infections, and lessen financial impacts. Developing an optimal sampling strategy for monitoring livestock for zoonotic diseases is vital for early detection. This approach ensures efficient resource use and scientifically credible results through random and representative sampling. Enhancing surveillance efforts enables proactive responses to disease threats, promoting livestock welfare and safeguarding public health.

The fulcrum of One Health is surveillance, which is a critical tool in our ability to anticipate, prevent, detect, and control diseases or events that can disrupt the balance in the biosphere (6–9). A scientifically designed surveillance plan is essential for epidemiological investigations when monitoring diseases in a population. The primary goal is to select representative animals to obtain unbiased and accurate estimates of disease frequency with minimal standard error. Common sampling methods include simple random sampling, systematic sampling, and stratified sampling, often with each animal serving as a sampling unit. Appropriate sample size determination considers factors such as regional disease prevalence, meta-analysis findings, diagnostic test parameters, and the desired confidence level.

In the absence of reliable prior data, a systematic review and meta-analysis provides a scientifically acceptable avenue for estimating sample sizes. A systematic review aims to comprehensively gather all relevant evidence related to specific research questions. In a meta-analysis, a common approach is to average the estimates of a comparable parameter from each study. Through meta-analysis, more precise estimates of treatment effects, risk factors for diseases, and other outcomes can be obtained compared to individual studies contributing to the pooled analysis (10). We aimed to establish a robust plan for estimating the prevalence of selected zoonotic diseases and transboundary diseases in India, based on a systematic review and meta-analysis.

Numerous epidemiological studies have reported the prevalence of individual zoonotic diseases and TADs in India, but these studies are geographically fragmented, employ different diagnostic methods, and often use inconsistent sampling strategies, making their findings difficult to compare or translate into a unified national surveillance programme. Furthermore, no study has developed an evidence-based sampling framework that simultaneously integrates multiple priority zoonotic diseases and TADs using pooled prevalence estimates derived from systematic reviews and meta-analyses. This represents a significant gap in the implementation of standardised national disease surveillance. To address this gap, the present study aimed to establish a scientifically rigorous sampling framework for estimating the prevalence of 10 priority zoonotic diseases and four TADs across India. The study integrates systematic review, random-effects meta-analysis, diagnostic test characteristics, and a two-stage stratified random sampling design to generate standardised surveillance sample sizes.

## 2 Materials and methods

### 2.1 Definitions

#### 2.1.1 Surveillance system, unit of surveillance, and surveillance system sensitivity

A surveillance system is a comprehensive set of procedures for collecting and interpreting information about a disease within a specific population. It consists of various components, each targeting specific surveillance units. For example, the surveillance units for on-farm testing, abattoir surveillance, and disease hotlines are individual animals, carcasses, and phone reports, respectively. A Unit of Surveillance refers to the average probability that a chosen surveillance unit will yield a positive outcome, assuming that the disease prevalence is equal to or higher than the specified design prevalence. A Surveillance System Sensitivity (SSe) is the average probability that the entire surveillance system will produce a positive surveillance outcome under the same conditions.

#### 2.1.2 Design and cluster-level prevalence

Cluster-level design prevalence refers to a prevalence value assigned to groups like villages, herds, or households, while unit-level design prevalence applies to individual entities such as animals, birds, or humans. Unit-level prevalence can be assessed within clusters (herds, flocks, or villages) across broader, unclustered populations, including animal/human populations or wildlife.

#### 2.1.3 Confidence level and herd sensitivity

The confidence level (95%) applied in the surveillance plan reflects the probability of detecting at least one positive sample if the disease is present at or above the specified design prevalence, and serves as a proxy for herd-level sensitivity (HSe). HSe is the probability that an infected herd/flock yields at least one positive result in the screening test, accounting for the diagnostic sensitivity (Se) of the assay used, given that the herd/flock is infected at or above the design prevalence.

#### 2.1.4 Sampling scheme

In this report, we utilised stratified random sampling, with village as the stratification variable for the targeted animal populations across different states. This approach incorporated reported (hence predetermined) sensitivity and specificity values for the tests employed for each disease, along with confidence intervals. A two-stage stratified random surveillance plan was generated with a 95% confidence level, using an in-house epi-calculator (11) which was originally developed under the National Animal Disease Referral Expert System version 2 (NADRES v2) of the Indian Council of Agricultural Research – National Institute of Veterinary Epidemiology and Disease Informatics (ICAR-NIVEDI), Bengaluru.

#### 2.1.5 Sampling frame and plan

The sampling frame is a list of entities (such as animals and humans) within the defined population and serves as the basis for sampling. Estimating the prevalence involves investigating a population or subpopulation where the presence of a pathogenic agent or disease, or exposure to a pathogenic agent can be detected (12). Baseline prevalence data can form the foundation of control programmes, including future surveillance programmes. The frequency and type of surveillance depend on the epidemiology of the agent or disease and desired outcomes. This study aims to demonstrate the practical application of R and epiR, in designing programmes to estimate disease prevalence and providing a quantitative basis for claims about pervasiveness of, or freedom from disease, and exploring how disease frequency varies by individual animals, place, and period.

### 2.2 Systematic review and meta-analysis

A comprehensive literature search was conducted in Google Scholar, ResearchGate, Krishikosha, PubMed, and ScienceDirect up to 2021. Search strings combined disease names with terms including “prevalence,” “epidemiology,” “India,” “systematic review,” and “cross-sectional study.” Full disease-specific search strings for all the 14 diseases are provided in Supplementary Table S1. Duplicate records were removed using Zotero, and titles, abstracts, and full texts were screened independently following the PRISMA guidelines (13). Studies were included if they reported original prevalence data from India, clearly described the study population and diagnostic methods, and provided sufficient data for prevalence estimation. Review articles, case reports, experimental studies, duplicate publications, and studies lacking adequate prevalence information were excluded. Study quality was independently assessed by two reviewers using predefined eligibility criteria. Inter-rater agreement, heterogeneity statistics (I^2^), and publication bias assessments were performed before pooling prevalence estimates (25). Disease-specific pooled prevalence estimates were generated using a random-effects meta-analysis because considerable between-study heterogeneity was anticipated owing to differences in geographical regions, study populations, sampling strategies, and diagnostic methods (14).

### 2.3 Sample plan

Sample size calculations were performed using the in-house epi-calculator of ICAR-NIVEDI, implementing a two-stage stratified random sampling design. Villages were considered the primary sampling units, while animals within villages constituted the secondary sampling units. Sample size estimation incorporated pooled prevalence obtained from the meta-analysis, diagnostic sensitivity and specificity, desired surveillance confidence level (95%), and design prevalence. Sample size calculations followed the two-stage cluster-sampling formulae of Cameron and Baldock (15, 16) for detecting disease at a specified design prevalence, adjusted for imperfect diagnostic sensitivity and specificity and for finite population correction at both the village and animal levels, as implemented in the epi-calculator. The software calculated both the required number of villages and the number of animals to be sampled per village. The objective of the sampling framework was to determine the number of villages and animals required to detect each disease within a two-stage surveillance design, using the meta-analysis-derived pooled prevalence as the design-stage prevalence input, at a specified target system sensitivity (SSe) and 95% surveillance confidence.

### 2.4 Inputs required

The following inputs are required to generate a prevalence plan:

- Unit-level design prevalence as either a proportion or an integer number of units;
- Cluster-level (herd level) design prevalence as either a proportion or an integer number of clusters;
- The estimated sensitivity and specificity for the test being employed;
- The target system sensitivity (SSe), which is the probability of detecting the disease if it is present at the specified design prevalence;
- Sampling frame data for all clusters in the population, including cluster-identification (labelled “Cluster ID”) and cluster size (labelled “Cluster Size”). Any clusters with a cluster size < 1 or where cluster size is missing were excluded from the calculations.

## 3 Results

Meta-analysis estimates the combined effect, giving more weight to more precise studies. We used a random-effects model to estimate the prevalence rate of 14 diseases (brucellosis, tuberculosis, Japanese encephalitis, Crimean-Congo haemorrhagic fever, coxiellosis/Q-fever, scrub typhus, cysticercosis, cryptosporidiosis, salmonellosis, listeriosis, lumpy skin disease, swine influenza, porcine reproductive and respiratory syndrome, and African swine fever).

Two junior reviewers (AM and PA) rated faced validity, and two senior reviewers (KPS & NRH) rated content validity, both using a five-point Likert scale across the 14 diseases under consideration. Each article’s final score was the average of its two ratings. Agreement between raters was assessed with quadratic-weighted Cohen’s kappa (k; Table 1). Agreement ranged from substantial to almost perfect across the disease-specific assessments (k = 0.63 to 0.91), indicating good consistency between the two reviewers.

**Table 1:** Disease-wise inter-rater agreement for methodological quality and risk-of-bias assessment using quadratic-weighted Cohen’s kappa (k).

Disease	Overall weighted Kappa (k)	95% CI
Brucellosis	0.82	0.79–0.85
Coxiellosis/Q-fever	0.88	0.83–0.91
Cysticercosis	0.88	0.83–0.93
CCHF	0.82	0.75–0.88
Scrub typus	0.90	0.79–0.96
Swine influenza	0.91	0.75–0.97
Tuberculosis	0.82	0.76–0.87
Japanese encephalitis	0.79	0.71–0.86
Listeriosis	0.84	0.75–0.92
Cryptosporidiosis	0.68	0.60–0.77
Salmonellosis	0.74	0.62–0.84
PRRS	0.63	0.08–0.94
LSD	0.77	0.55–1.00
ASF	0.77	0.66–0.92

From various studies reported for the period 2000 to 2021 in India (Table 2), meta-analysis was employed to calculate the disease prevalence rates, which were devised for a surveillance plan. For scrub typhus and listeriosis, animal-reservoir prevalence data were insufficient in the Indian literature to derive a livestock-based design prevalence; pooled estimates for these two diseases were therefore derived from human and food-matrix studies, respectively (Table 2). Consequently, the corresponding sampling units differ from the remaining 12 diseases: for scrub typhus, sampling is directed at rodent/vector collection sites within the selected villages, and for listeriosis, at food product batches from processing and retail premises serving the same villages, rather than from live animals directly. The overall totals of 34,975 samples and 2,018 villages therefore include these non-livestock components, which are itemised by disease in Table 2. The outcomes of the analysis are presented in forest plots displaying the prevalence percentages for each of the diseases (Figures 1–14).

**Table 2:** Details of data used and the generated sampling plan at 95% confidence interval.

Disease	No. of Studies	Total Sample size	Overall prevalence (%)	Species to be covered	No. of sampled villages	No. of samples	No. of samples per village/herd
Brucellosis	228	101,065	12	C, B, S, G, P	160	3,228	18–21
Coxiellosis/Q-fever	42	7,557	10	C, B	185	4,517	21–25
Cysticercosis	64	35,409	18	P	152	2,205	13–15
CCHF	38	11,617	11	C, B, G	154	3,695	20–23
Scrub typus	11	3,853	22	Rodents*	134	1,553	11–12
Swine influenza	5	9,400	30	P	105	1,075	09–10
Tuberculosis	30	27,329	12	C, B	158	3,230	18–21
Japanese encephalitis	49	32,653	14	P	134	2,389	16–18
Listeriosis	24	8,084	9	Foodstuff^$^	156	4,183	23–27
Cryptosporidiosis	24	8,292	27	C, B (calves)	116	1,161	09–10
Salmonellosis	25	5,765	14	C, P, Ck, Dk	133	2,341	16–18
PRRS	4	3,886	29	P	128	1,173	8–9
LSD	3	3,093	18	C	157	2,250	13–15
ASF	5	5,478	19^#^	P	146	1975	12-14

*Pooled prevalence derived from human case data (owing to insufficient data from animals); sampling plan targets rodent/vector collection sites. ^$^ Pooled prevalence derived from food-matrix studies; sampling plan targets food product batches from processing and retail premises. ^#^Deduced from studies conducted from outside Asia, owing to the absence of India-specific data at the time of initiation of this study. Abbreviations: ASF, African swine fever; B, buffalo; C, cattle; CCHF, Crimean-Congo haemorrhagic fever; Ck, chicken; Dk, duck; G, goat; LSD, lumpy skin disease; P, pig; PRRS, porcine reproductive and respiratory syndrome; S, sheep.

**Figure 1:**
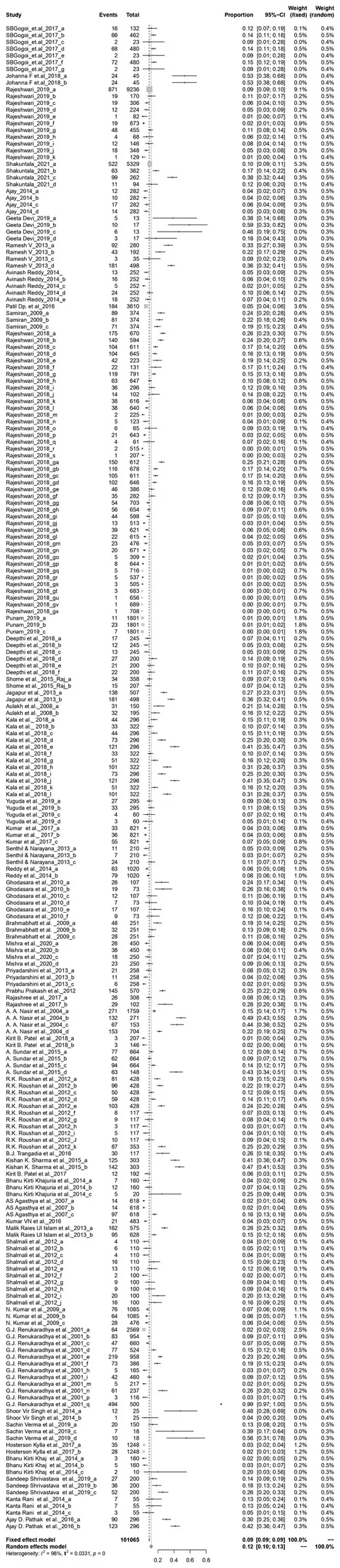
Meta-analysis and forest plots for Brucellosis. Literature from various sources was collated. The data were extracted, and the quality of the data was assessed before modelling to estimate prevalence rates. A meta-analysis was performed to combine the results. The forest plots represent individual study results as well as pooled estimates. Each square represents a study, with its size reflecting the sample size. The horizontal line within each square represents the confidence interval. The diamond at the bottom summarises the pooled estimates from all studies.

**Figure 2:**
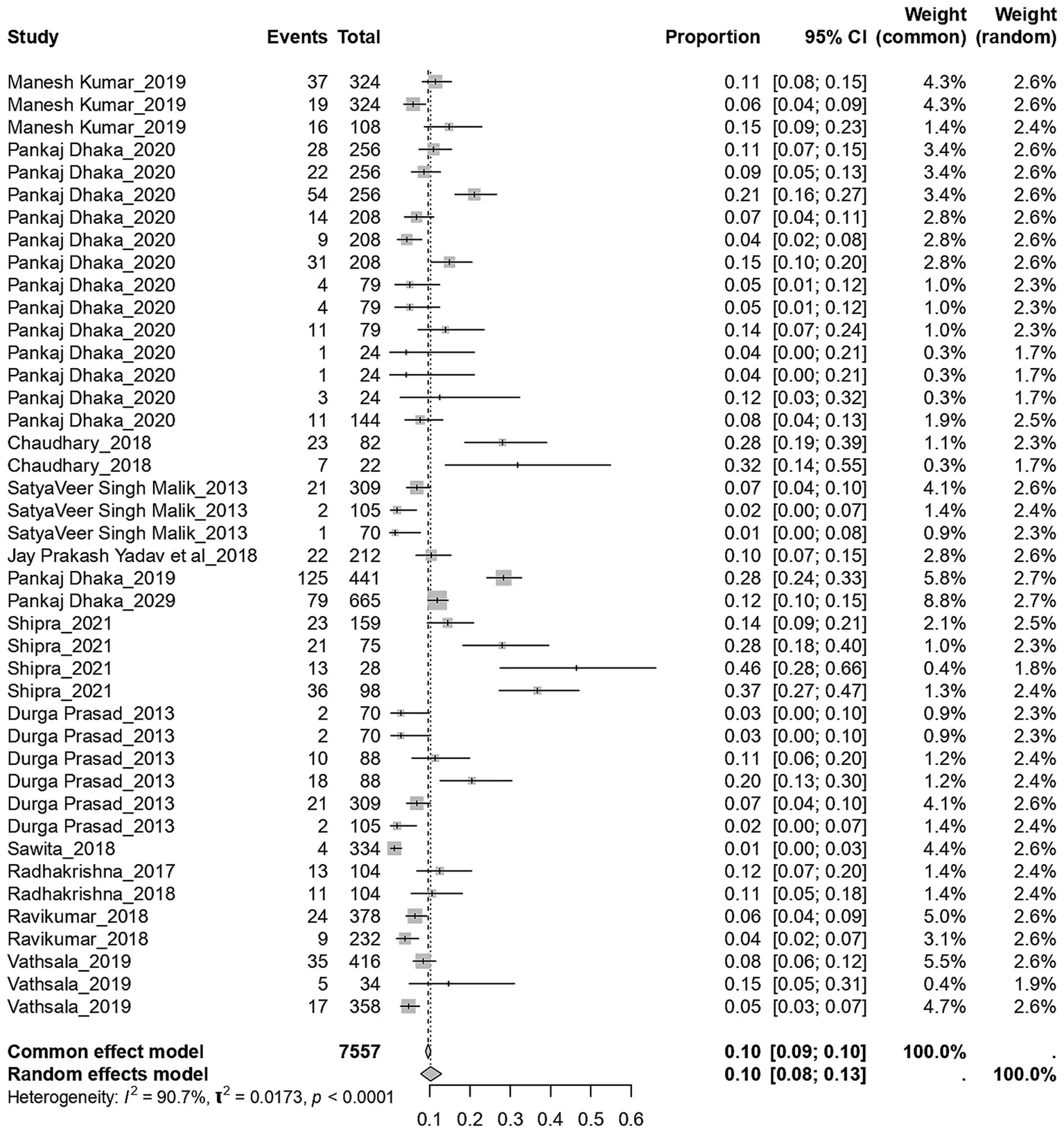
Meta-analysis and forest plots for Tuberculosis. Literature from various sources was collated. The data were extracted, and the quality of the data was assessed before modelling to estimate prevalence rates. A meta-analysis was performed to combine the results. The forest plots represent individual study results as well as pooled estimates. Each square represents a study, with its size reflecting the sample size. The horizontal line within each square represents the confidence interval. The diamond at the bottom summarises the pooled estimates from all studies.

**Figure 3:**
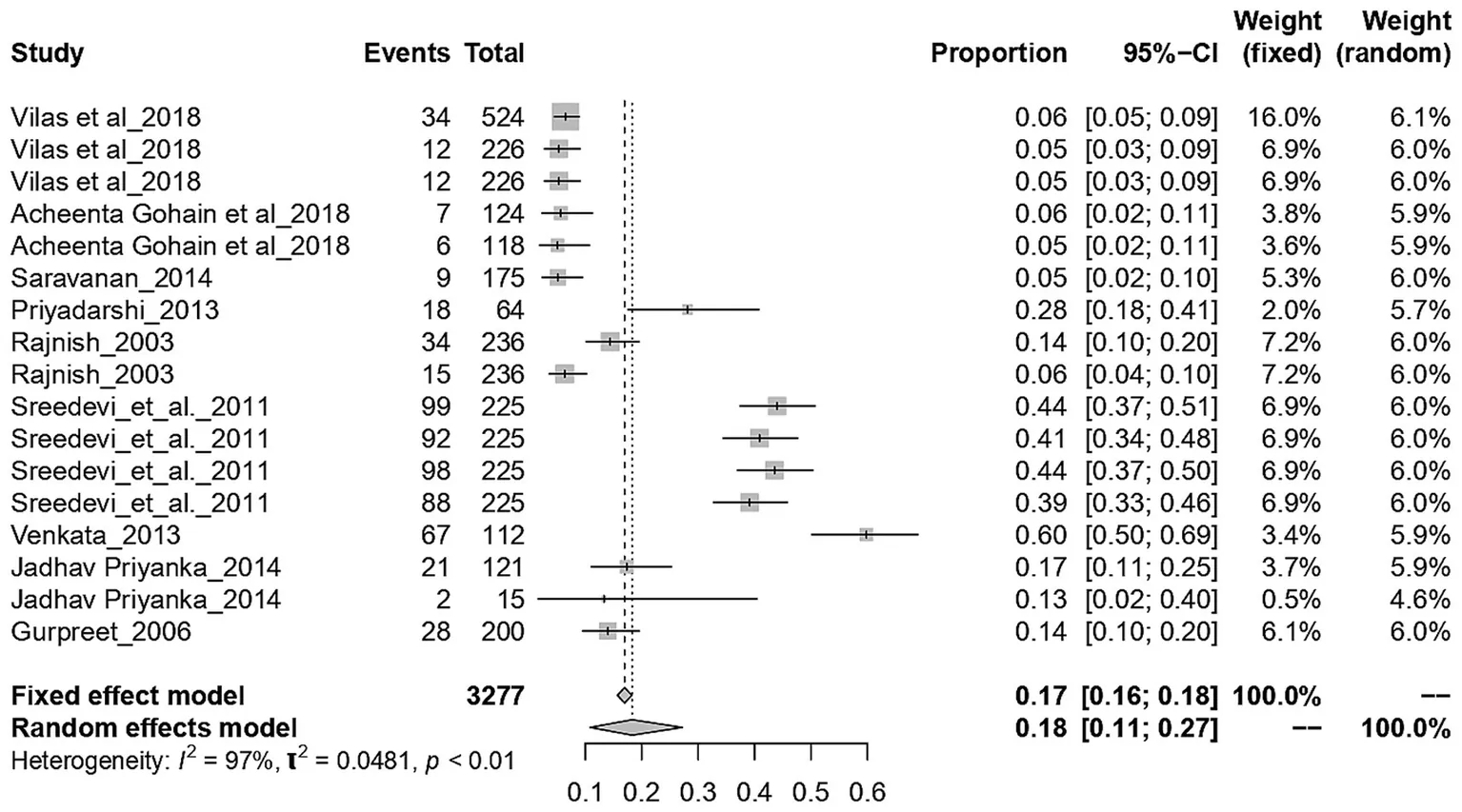
Meta-analysis and forest plots for Japanese encephalitis (JE). Literature from various sources was collated. The data were extracted, and the quality of the data was assessed before modelling to estimate prevalence rates. A meta-analysis was performed to combine the results. The forest plots represent individual study results as well as pooled estimates. Each square represents a study, with its size reflecting the sample size. The horizontal line within each square represents the confidence interval. The diamond at the bottom summarises the pooled estimates from all studies.

**Figure 4:**
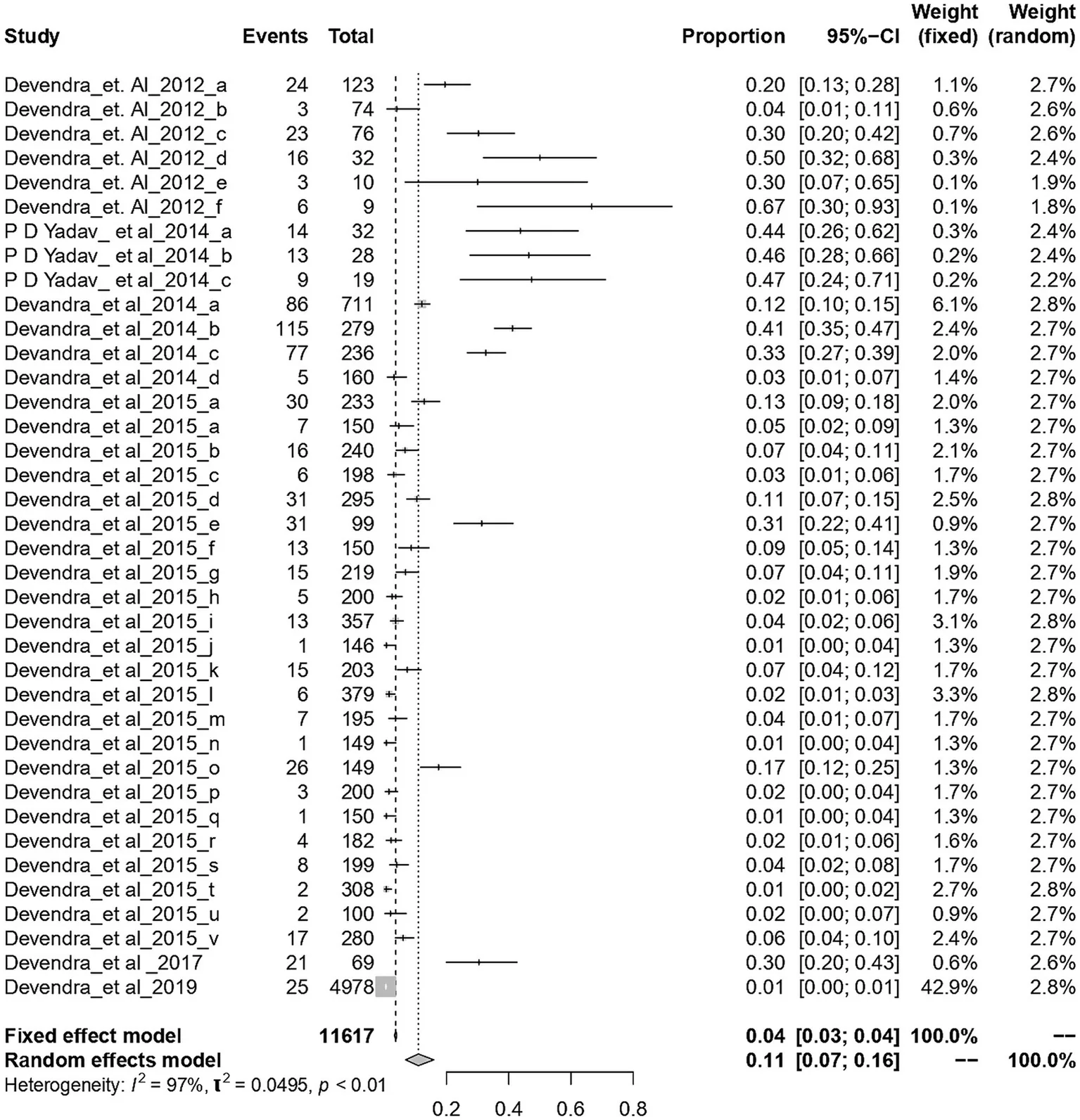
Meta-analysis and forest plots for Crimean-Congo haemorrhagic fever (CCHF). Literature from various sources was collated. The data were extracted, and the quality of the data was assessed before modelling to estimate prevalence rates. A meta-analysis was performed to combine the results. The forest plots represent individual study results as well as pooled estimates. Each square represents a study, with its size reflecting the sample size. The horizontal line within each square represents the confidence interval. The diamond at the bottom summarises the pooled estimates from all studies.

**Figure 5:**
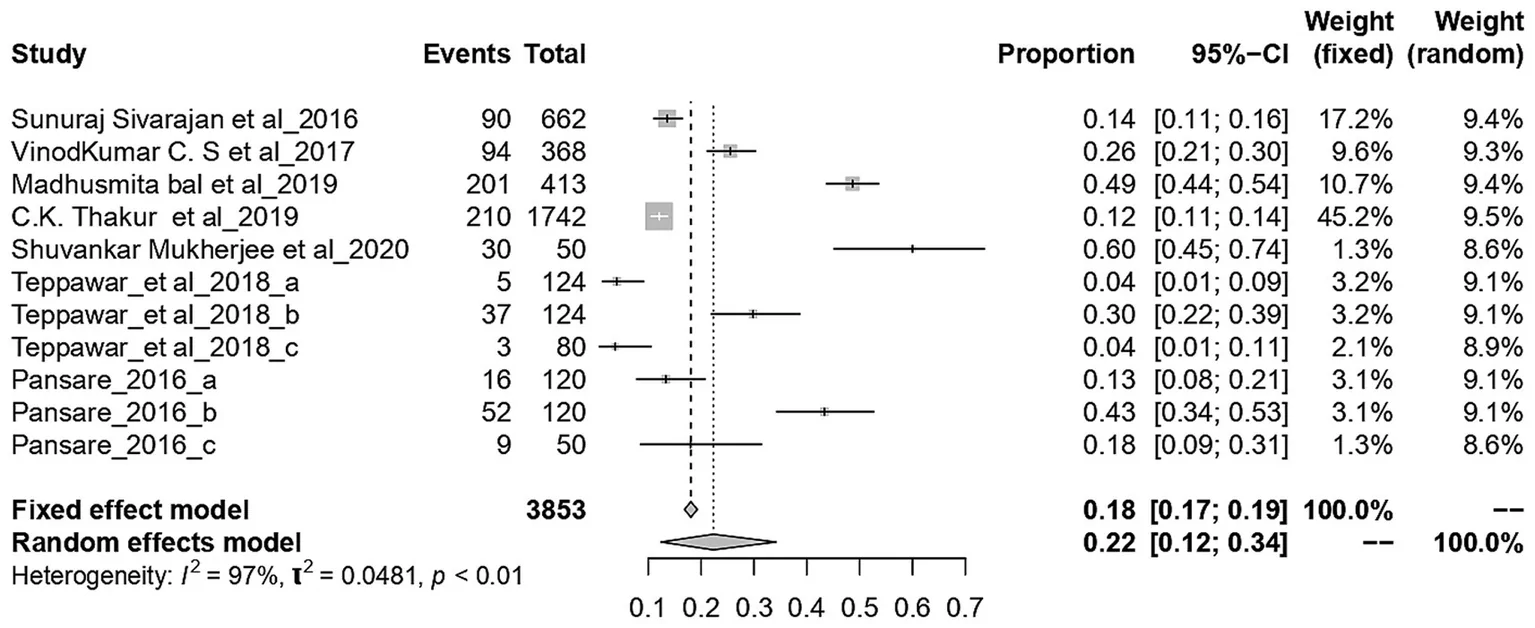
Meta-analysis and forest plots for Coxiellosis (Q fever). Literature from various sources was collated. The data were extracted, and the quality of the data was assessed before modelling to estimate prevalence rates. A meta-analysis was performed to combine the results. The forest plots represent individual study results as well as pooled estimates. Each square represents a study, with its size reflecting the sample size. The horizontal line within each square represents the confidence interval. The diamond at the bottom summarises the pooled estimates from all studies.

**Figure 6:**
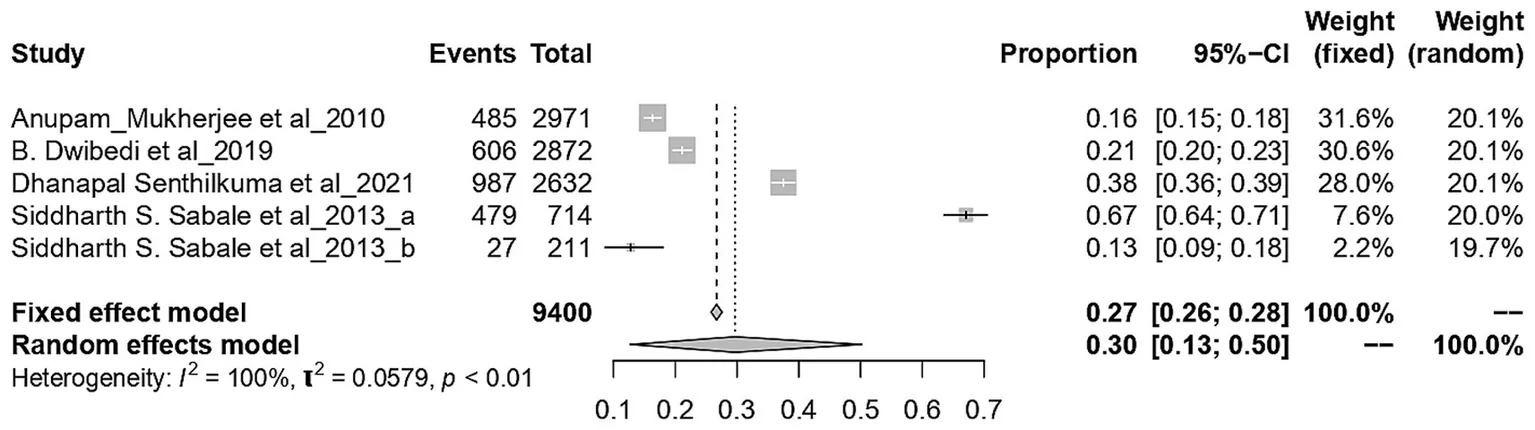
Meta-analysis and forest plots for Scrub Typhus. Literature from various sources was collated. The data were extracted, and the quality of the data was assessed before modelling to estimate prevalence rates. A meta-analysis was performed to combine the results. The forest plots represent individual study results as well as pooled estimates. Each square represents a study, with its size reflecting the sample size. The horizontal line within each square represents the confidence interval. The diamond at the bottom summarises the pooled estimates from all studies.

**Figure 7:**
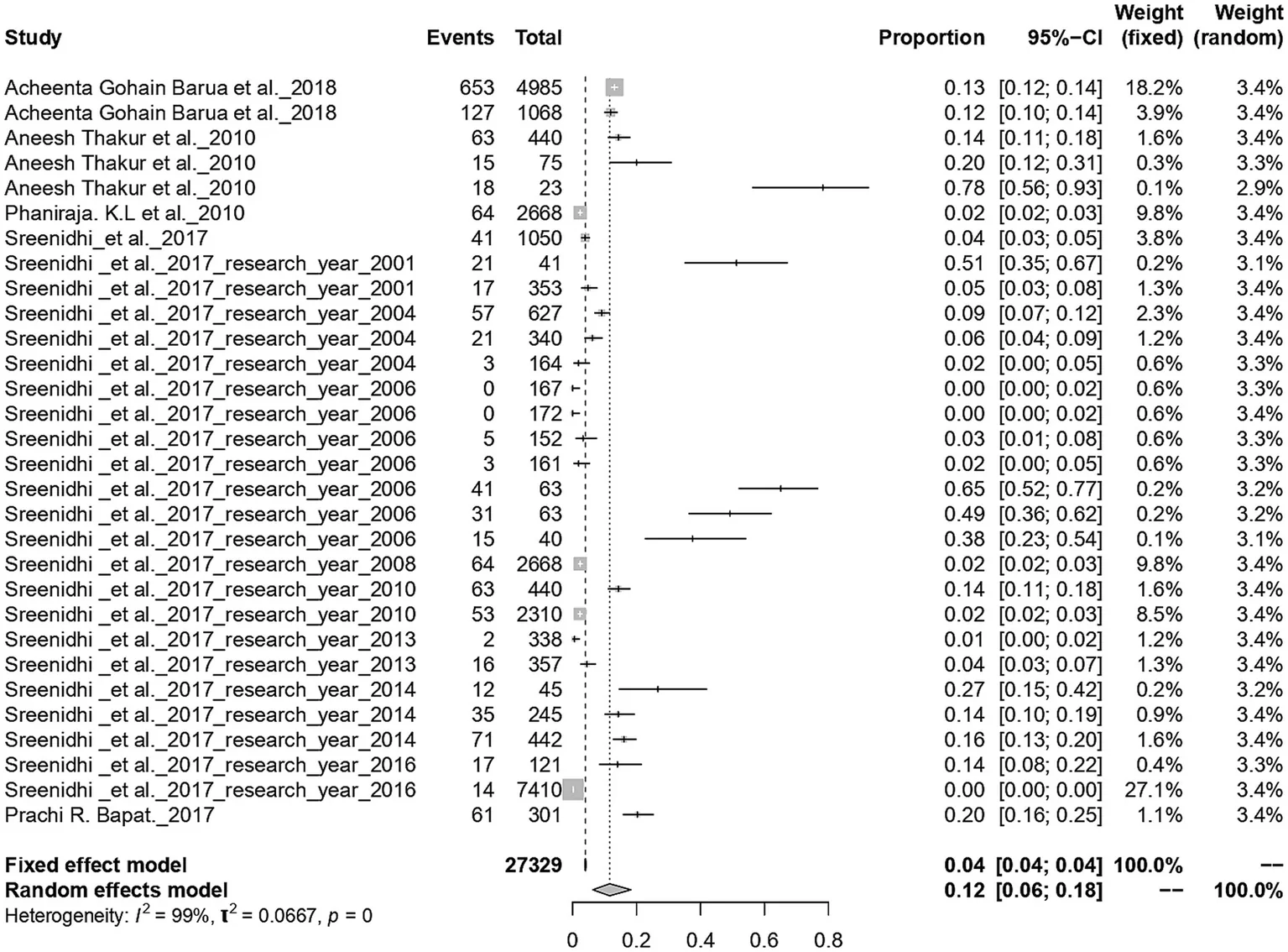
Meta-analysis and forest plots for Cryptosporidiosis. Literature from various sources was collated. The data were extracted, and the quality of the data was assessed before modelling to estimate prevalence rates. A meta-analysis was performed to combine the results. The forest plots represent individual study results as well as pooled estimates. Each square represents a study, with its size reflecting the sample size. The horizontal line within each square represents the confidence interval. The diamond at the bottom summarises the pooled estimates from all studies.

**Figure 8:**
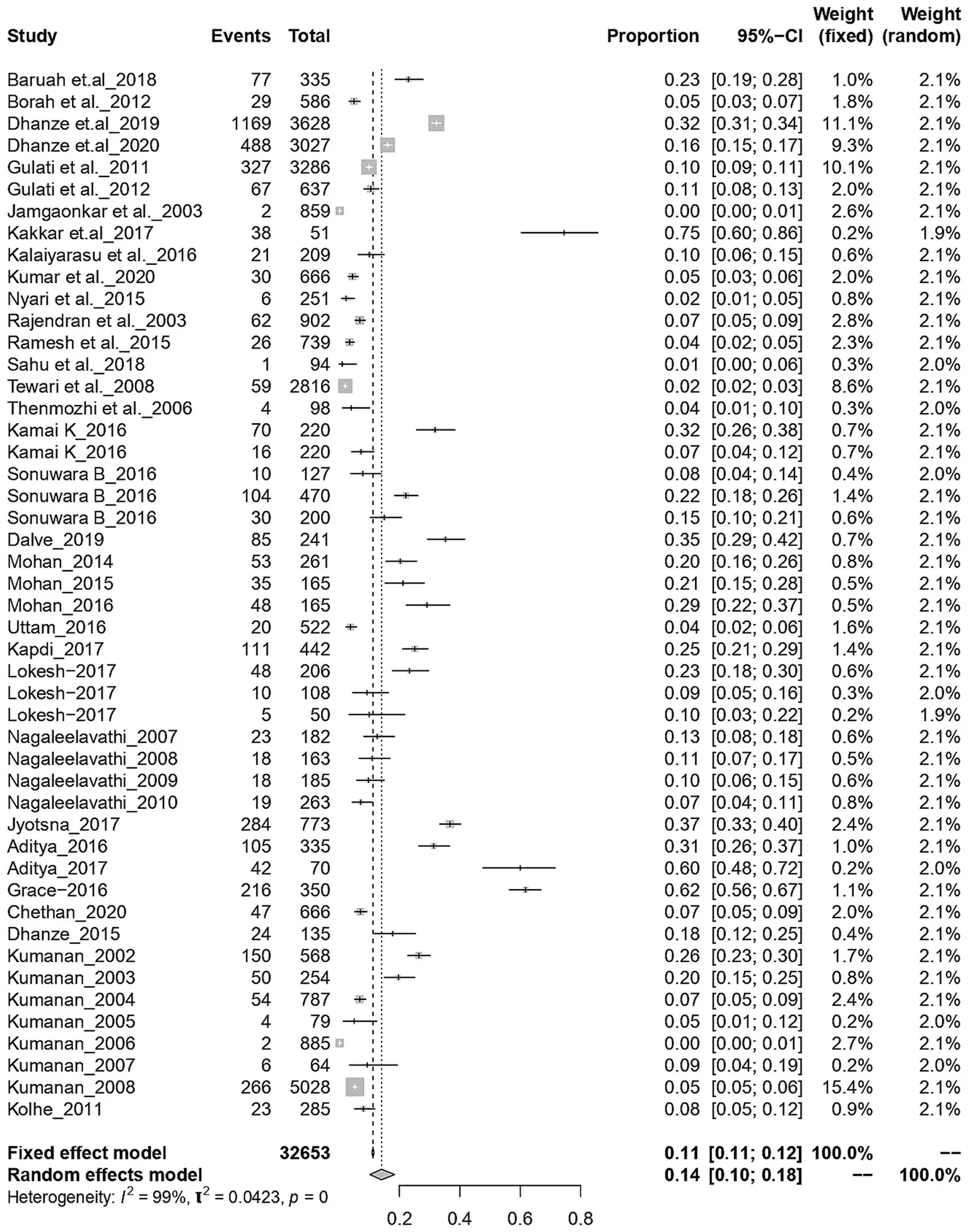
Meta-analysis and forest plots for Cysticercosis. Literature from various sources was collated. The data were extracted, and the quality of the data was assessed before modelling to estimate prevalence rates. A meta-analysis was performed to combine the results. The forest plots represent individual study results as well as pooled estimates. Each square represents a study, with its size reflecting the sample size. The horizontal line within each square represents the confidence interval. The diamond at the bottom summarises the pooled estimates from all studies.

**Figure 9:**
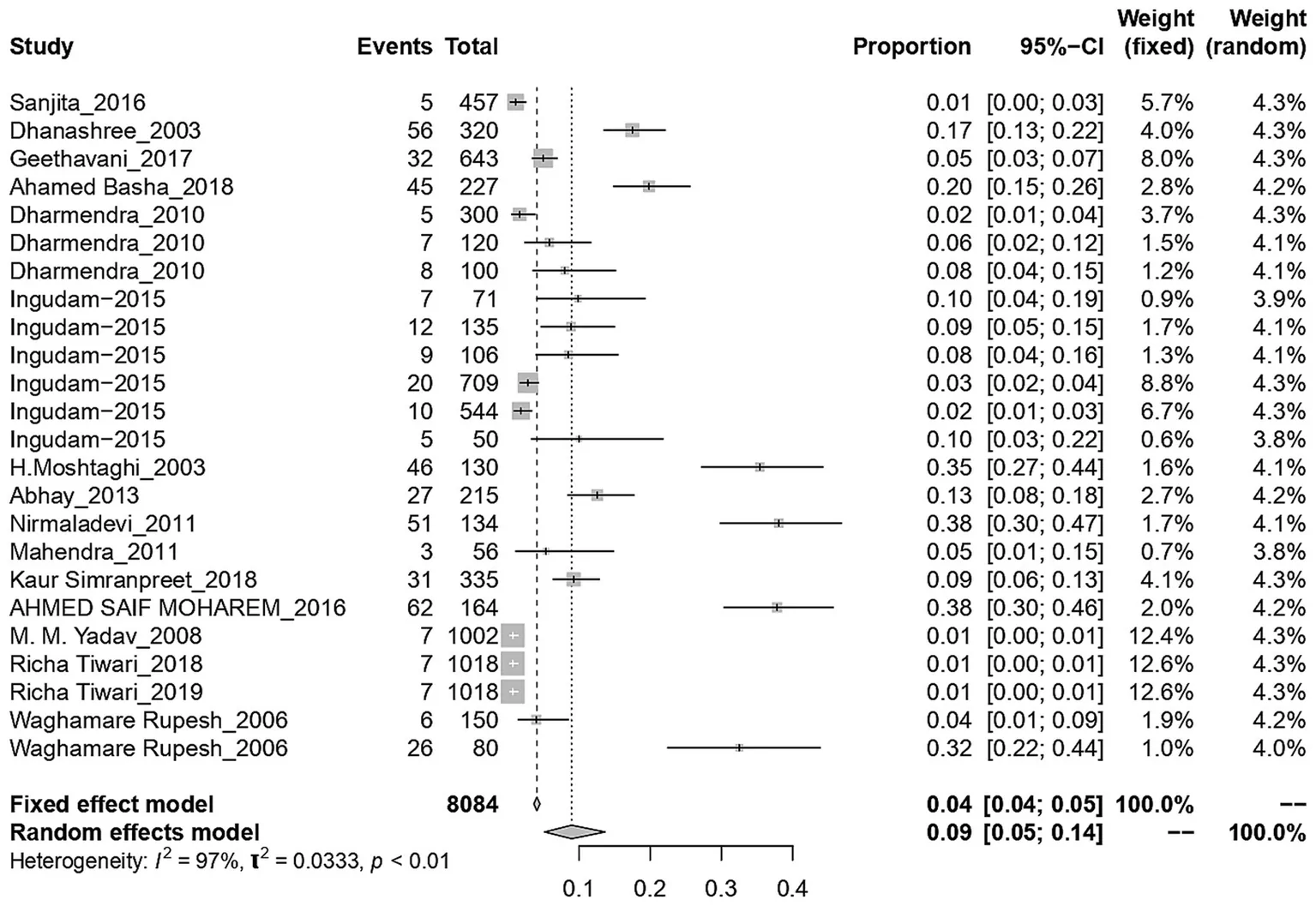
Meta-analysis and forest plots for Salmonellosis. Literature from various sources was collated. The data were extracted, and the quality of the data was assessed before modelling to estimate prevalence rates. A meta-analysis was performed to combine the results. The forest plots represent individual study results as well as pooled estimates. Each square represents a study, with its size reflecting the sample size. The horizontal line within each square represents the confidence interval. The diamond at the bottom summarises the pooled estimates from all studies.

**Figure 10:**
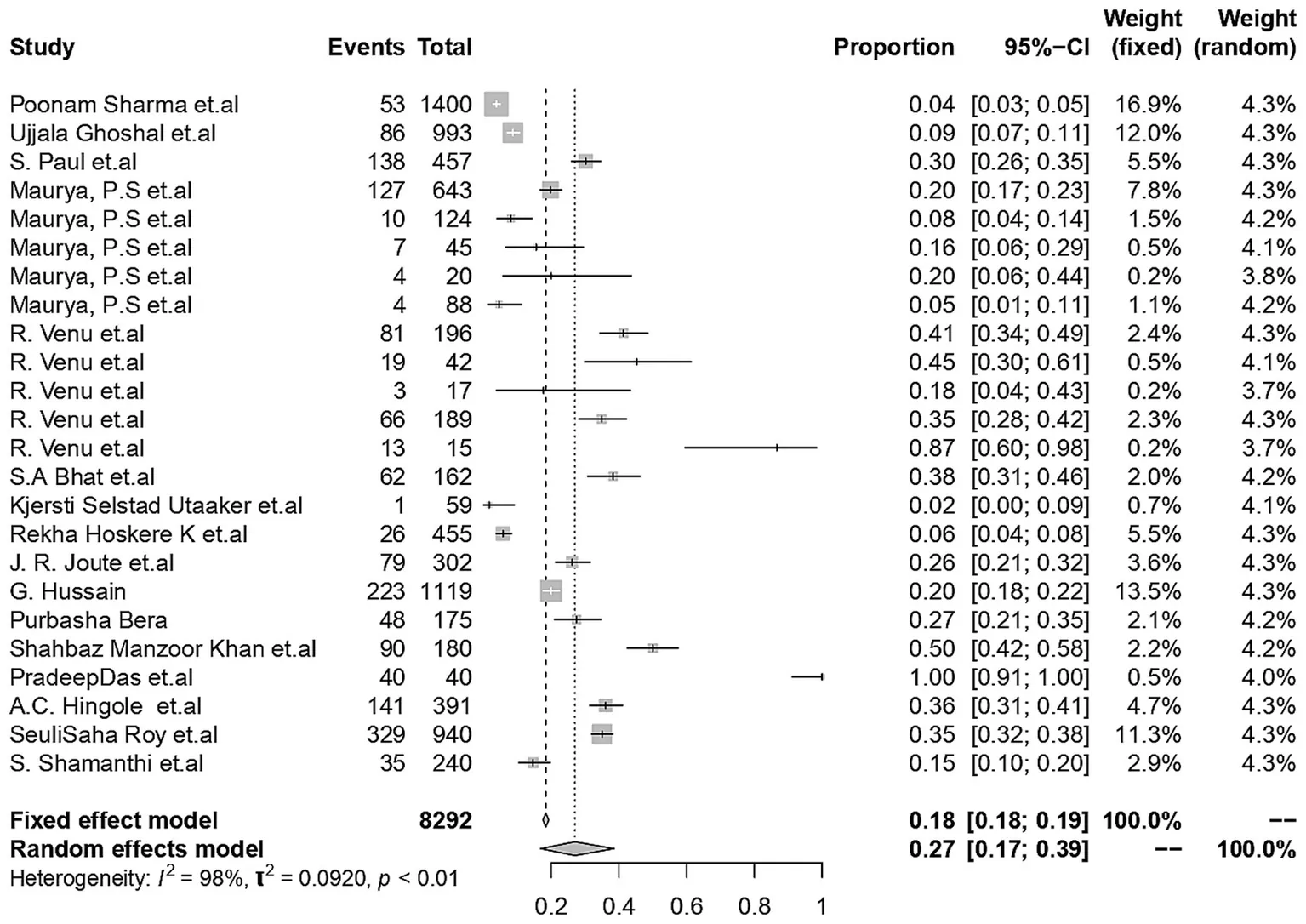
Meta-analysis and forest plots for Listeriosis. Literature from various sources was collated. The data were extracted, and the quality of the data was assessed before modelling to estimate prevalence rates. A meta-analysis was performed to combine the results. The forest plots represent individual study results as well as pooled estimates. Each square represents a study, with its size reflecting the sample size. The horizontal line within each square represents the confidence interval. The diamond at the bottom summarises the pooled estimates from all studies.

**Figure 11:**
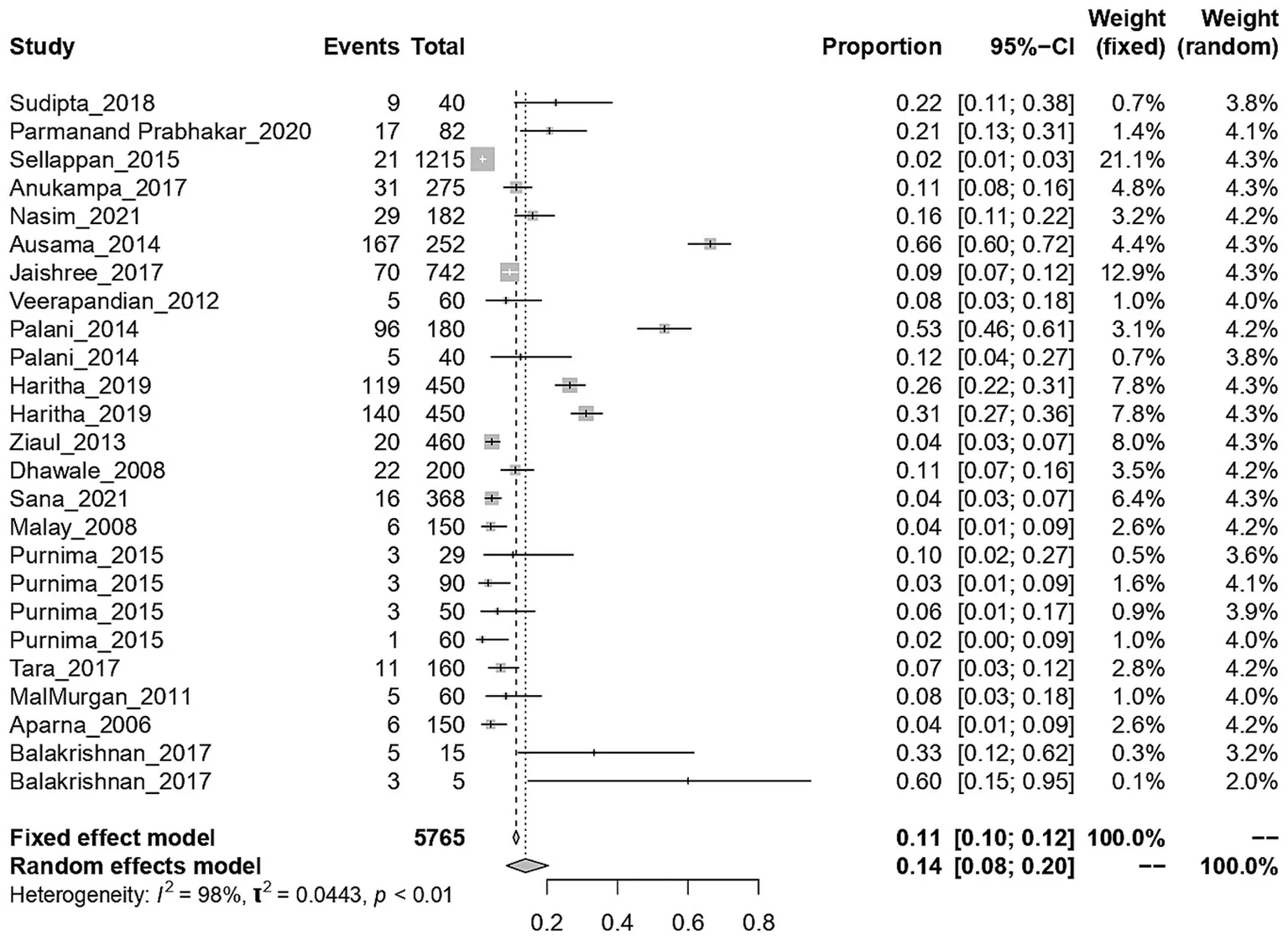
Meta-analysis and forest plots for Lumpy skin disease. Literature from various sources was collated. The data were extracted, and the quality of the data was assessed before modelling to estimate prevalence rates. A meta-analysis was performed to combine the results. The forest plots represent individual study results as well as pooled estimates. Each square represents a study, with its size reflecting the sample size. The horizontal line within each square represents the confidence interval. The diamond at the bottom summarises the pooled estimates from all studies.

**Figure 12:**
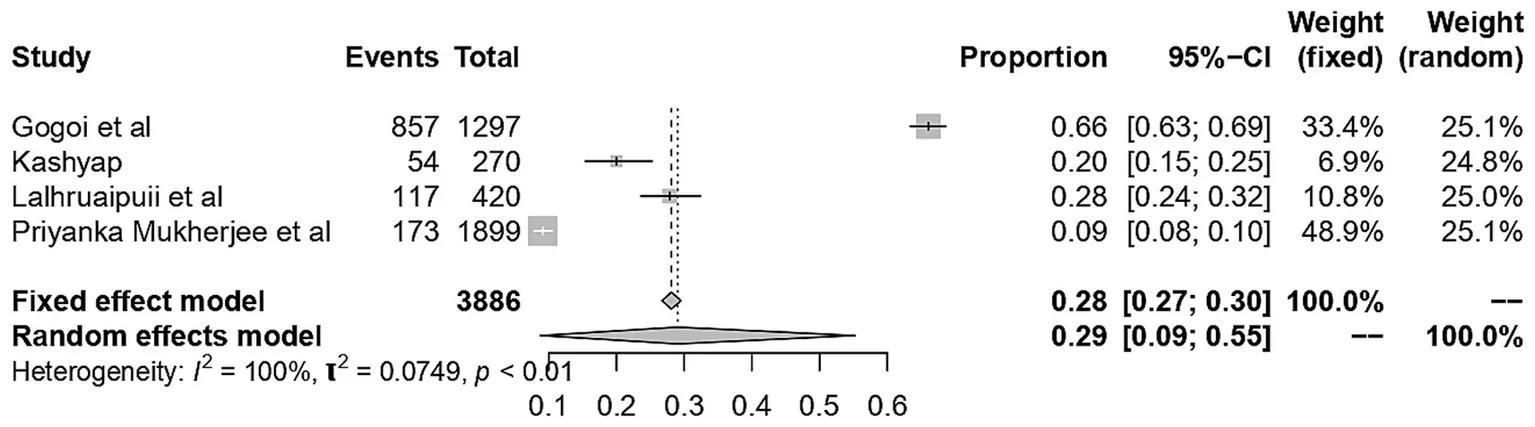
Meta-analysis and forest plots for Swine influenza. Literature from various sources was collated. The data were extracted, and the quality of the data was assessed before modelling to estimate prevalence rates. A meta-analysis was performed to combine the results. The forest plots represent individual study results as well as pooled estimates. Each square represents a study, with its size reflecting the sample size. The horizontal line within each square represents the confidence interval. The diamond at the bottom summarises the pooled estimates from all studies.

**Figure 13:**
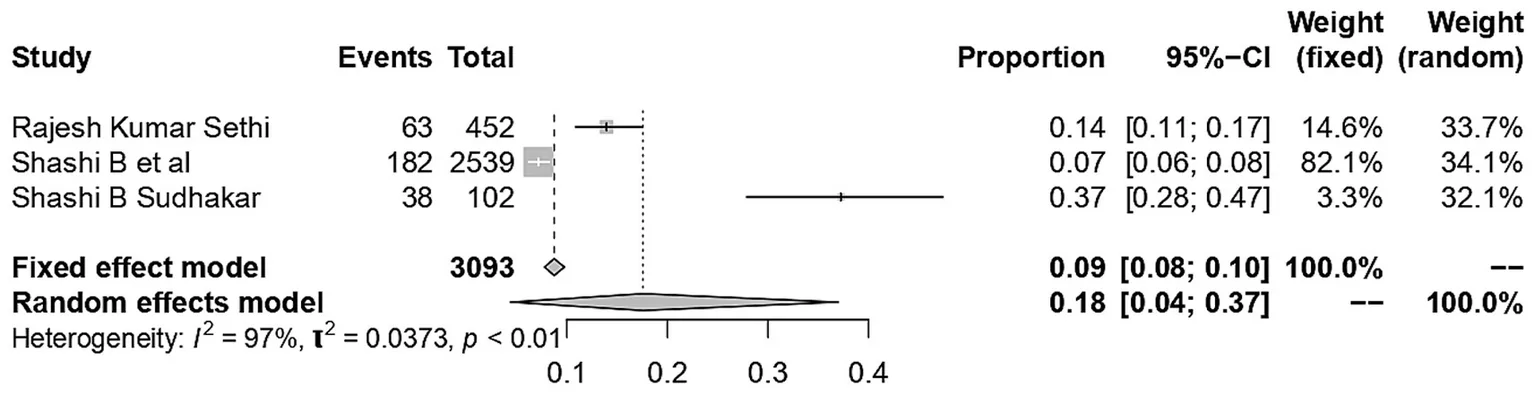
Meta-analysis and forest plots for Porcine reproductive and respiratory syndrome (PRRS). Literature from various sources was collated. The data were extracted, and the quality of the data was assessed before modelling to estimate prevalence rates. A meta-analysis was performed to combine the results. The forest plots represent individual study results as well as pooled estimates. Each square represents a study, with its size reflecting the sample size. The horizontal line within each square represents the confidence interval. The diamond at the bottom summarises the pooled estimates from all studies.

**Figure 14:**
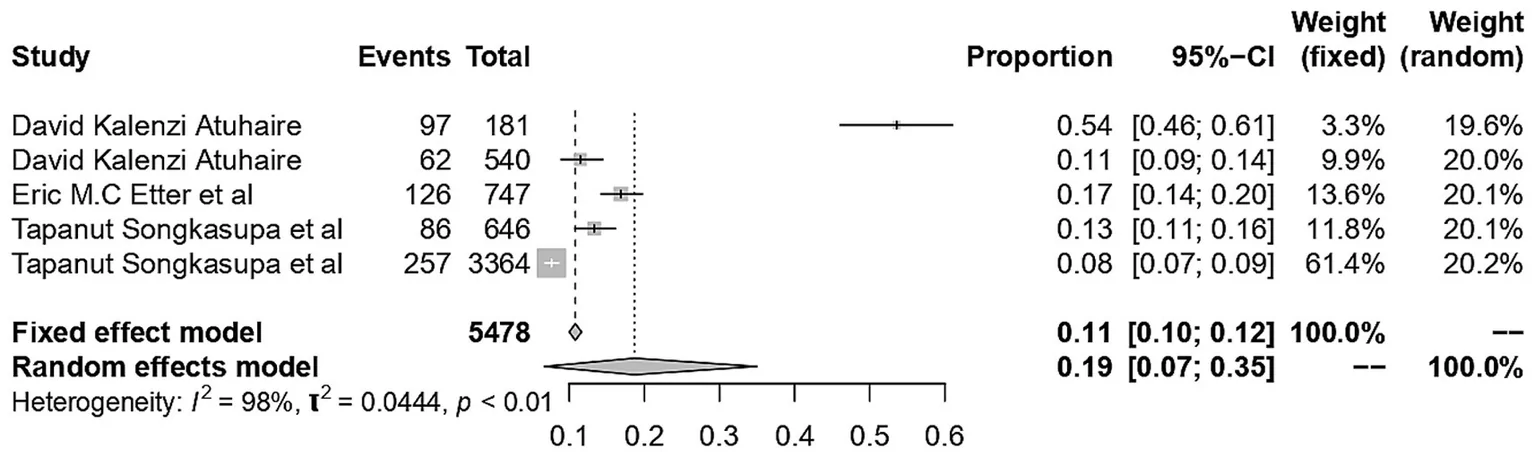
Meta-analysis and forest plots for African swine fever (ASF). Literature from various sources was collated. The data were extracted, and the quality of the data was assessed before modelling to estimate prevalence rates. A meta-analysis was performed to combine the results. The forest plots represent individual study results as well as pooled estimates. Each square represents a study, with its size reflecting the sample size. The horizontal line within each square represents the confidence interval. The diamond at the bottom summarises the pooled estimates from all studies.

Pooled prevalence and Heterogeneity (I^2^) for each disease are summarised in Table 3. Substantial between-study heterogeneity was observed across all 14 diseases, with I^2^ values ranging from 91% for Q-fever to 100% for swine influenza and porcine reproductive and respiratory syndrome (PRRS), and heterogeneity was statistically significant (*p* < 0.01) for every disease evaluated. The corresponding τ^2^ estimates, reflecting the magnitude of true between-study variance, ranged from 0.0168 (Q-fever) to 0.1400 (cryptosporidiosis). This high and consistent heterogeneity across diseases justified the use of a random-effects model for pooling prevalence estimates, as differences in geographical regions, study populations, sampling strategies, and diagnostic methods were expected to contribute meaningfully to variation in reported prevalence.

**Table 3:** Pooled prevalence and Heterogeneity summary for the 14 diseases.

Disease	Pooled prevalence, % (95% CI)	Heterogeneity (I^2^; %)	τ^2^	*p* (heterogeneity)
Brucellosis	12 (10–13)	98	0.0331	0
Coxiellosis/Q-fever	10 (8–13)	91	0.0168	<0.01
Cysticercosis	4 (3–7)	97	0.0310	0
CCHF	11 (7–16)	97	0.0495	<0.01
Scrub typhus	22 (12–34)	97	0.0481	<0.01
Swine influenza	30 (13–50)	100	0.0423	<0.01
Tuberculosis	12 (6–18)	99	0.0667	0
Japanese encephalitis	14 (10–18)	99	0.0423	0
Listeriosis	9 (5–14)	97	0.0333	<0.01
Cryptosporidiosis	27 (17–39)	98	0.1400	<0.01
Salmonellosis	14 (8–20)	98	0.0443	<0.01
PRRS	29 (9–55)	100	0.0749	<0.01
LSD	18 (4–37)	97	0.0373	<0.01
ASF	19 (7–35)	98	0.0444	<0.01

Formulating the sampling plan involved calculations to determine the number of sampled villages, the total samples per state, and the distribution of samples within each village (Figure 15). Table 2 reflects the sampling plan generated through meta-analysis and a two-stage stratified random sampling.

**Figure 15:**
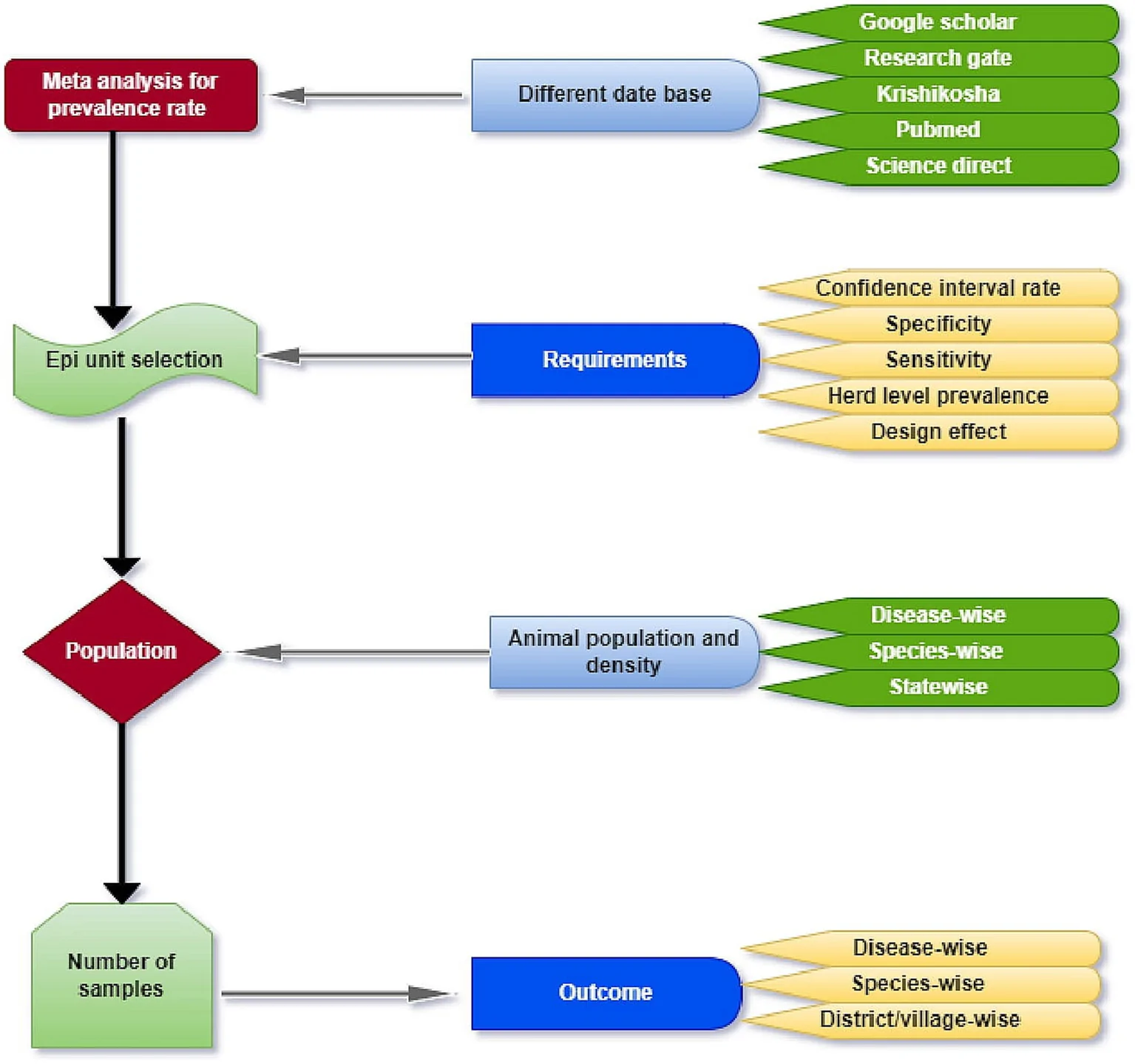
Schema used for deriving the sample numbers.

Based on the prevalence rates and a stratified random sampling with the village as a stratification variable, we devised a sampling plan that includes predetermined test sensitivity and specificity along with confidence intervals. This provides guidelines for selecting the villages and animals to be sampled, calculated based on the targeted population. For instance, for brucellosis, the number of samples per village ranged from 18 to 21, estimated based on the number of sampled villages and total samples collected for each state. The minimum number of samples projected (herd/flock) reflected the degree of confidence we have established. The confidence level indicates how likely the set assumptions are true, specifically the probability of finding at least one positive animal from those tested premises. It also serves as a proxy for herd sensitivity (HSe) in detecting the disease. The HSe is the probability that an infected herd/flock will yield a positive result, i.e., one or more samples are expected to be positive in the screening test (taking into account its specific diagnostic Se), given that the herd/flock is infected at a design prevalence equal to or greater than the set value.

## 4 Discussion

Zoonotic diseases pose a significant public health threat to India as the country is thought to be one of the global ‘hotspots’ due to the dense human and livestock population living in proximity, as well as sociocultural practices (3, 17). This is evident from the high rates of re-emergence of diseases associated with rapid socio-ecological and environmental changes (18). Establishing national One Health platforms and policies is therefore crucial to enhancing the coordination and integration of activities and programmes across different sectors. Although there is an increasing commitment to One Health worldwide, practical implementation remains challenging (5).

The primary objective in disease control or eradication is to promptly identify all infected or previously exposed animals within the target population. However, creating an exhaustive list (sampling frame) of each animal (sampling unit) in the population can be challenging. As a result, animals are often grouped into herds or flocks as the initial sampling unit. Therefore, any sampling plan should prioritise selecting the appropriate number of premises (sampling units) for each disease in question. Within these chosen premises, a sample of animals should be drawn from the entire animal population (sampling frame) in the herd or flock to determine their infection or exposure status.

The process of estimating the sample size for a disease involves calculating the number of premises or sites and the number of animals to be sampled (census-based). The alternative strategy employs a sample-based approach rather than a census-based one, albeit at a higher cost per test (19). This may include specific details about the actual premises and animals that will be sampled, as well as the methods used to select those premises and animals. To determine the number of premises to be sampled in each disease control area or zone, we conducted a systematic review and meta-analysis, which involved combining data from multiple research studies to obtain a comprehensive synthesis of information (20). These tools utilise statistical methods to gather and summarise research findings effectively. In our study, the results of the meta-analysis provided prevalence estimates, which are instrumental in devising a robust plan to estimate the prevalence of 14 diseases.

The sample consists of individuals selected from the target population through a specific sampling process in the study (21). Developing an effective sampling strategy for monitoring livestock diseases is crucial for early detection and optimising limited resources. This ensures that the results are scientifically valid, as the sampling is random, representative, and independent of the units selected. A surveillance system may have various components or activities, and their specific purposes should be explicitly stated (22). Those collecting samples must clearly define the problem or questions they want the analysis to address (23) and evaluate if their plan can yield the desired results and answers.

Sample size calculation is a critical aspect associated with statistical power, as larger sample sizes generally lead to increased study power. Ideally, a study should have a minimum power of 80% to ensure reliable results (24). Thus, determining the appropriate sample size is crucial and forms the foundation for designing a study protocol. Timely detection of disease is paramount, as it reduces the risk of spreading to new areas, minimises infections, and mitigates the financial impact. For surveillance programmes to be effective, prevalence data combined with outbreak data provide robust foundations to design sampling frames. Surveillance methods entail the voluntary reporting of cases by primary care providers and farmers to the veterinary health system, whereas active surveillance of livestock diseases involves periodic sampling and testing by veterinary health officials. Active surveillance methods often outperform passive methods in achieving targeted objectives and prove to be more cost-effective, especially for systems with limited resources.

During the planning stage of a study, researchers must decide whether to work with the entire target population or a sample. Opting for a sample involves several essential steps, such as estimating the sample size, identifying the sample frame, and selecting the appropriate sampling method (21). The minimum number of samples taken in each site (herd/flock) is determined based on the level of confidence, typically set at 95%. We also applied a 95% confidence interval, along with the sensitivity and specificity of the test, as well as a herd-level prevalence of 10 to 20%, depending on the specific pathogen, to derive the sample numbers.

Substantial heterogeneity (Table 2) was observed for most diseases, reflecting genuine differences across primary studies rather than sampling variation alone. Likely contributors include differences in diagnostic assays and their reported sensitivity and specificity, variation in target populations (e.g., organised vs. unorganised/backyard livestock systems), regional agro-ecological and management differences affecting exposure risk, and differences in study design (cross-sectional surveys vs. abattoir- or hospital-based sampling). Because pooled estimates were derived predominantly from convenience and purposive samples reported in the primary literature, rather than from a nationally representative baseline survey, they should be interpreted as design-stage estimates for sample-size planning rather than as definitive national prevalence figures.

The study’s results provide a statistically grounded sampling strategy for estimating the prevalence of livestock diseases, including zoonoses, that can support the effective utilisation of limited surveillance resources. The two-stage stratified random design is intended to yield representative and independent sampling units under the stated assumptions. These findings have direct implications for field implementation. States or regions contributing a disproportionate number of primary studies (e.g., for brucellosis and cysticercosis) may not be representative of India as a whole, and prevalence is likely to vary by agro-climatic zone, livestock management system, and species. We therefore recommend that state-level implementing agencies treat the sample sizes in Table 1 as minimum requirements and consider stratifying further by known risk factors where local epidemiological data permit.

Prompt detection of diseases can substantially mitigate the costs associated with disease eradication, illnesses among livestock and productivity losses, besides reducing disease and associated losses in humans in the case of zoonotic diseases. A fundamental objective in controlling and eradicating disease outbreaks is the swift identification of all infected or exposed animals within the target population. Robust surveillance plans, however, depend on strong baseline prevalence data for the disease(s) in question. The current study presents a plan to reinforce the surveillance activities for select zoonotic diseases and transboundary animal diseases, as a prelude to strengthening One Health approaches in India. A crucial prerequisite for formulating this plan is estimating the prevalence rate of each identified pathogen, a process accomplished through meta-analysis. The study’s results provide an optimal sampling strategy for estimating the prevalence of livestock diseases, including zoonosis. This strategy plays a pivotal role in developing robust surveillance strategies and ensures the effective utilisation of limited resources. Additionally, the plan guarantees the selection of random, representative, and independent sampling units, thus ensuring scientifically valid outcomes. Such approaches could herald transformative advancements in the country’s public health endeavours.

This study, however, has a few limitations that should be considered when interpreting and applying the proposed sampling plan. First, as noted above, the pooled prevalence estimates are subject to statistically significant heterogeneity for several diseases and should be regarded as planning estimates rather than definitive population parameters. Second, no India-specific field prevalence data were available for African swine fever (ASF) at the time of this review, since the disease was first reported in the country only in 2020; the design prevalence for ASF was therefore conservatively deduced from regional Asian data, pending accumulation of India-specific evidence. Third, pooled prevalence estimates for scrub typhus and listeriosis were derived from human and food-matrix studies, respectively, rather than from live-animal data, because sufficient animal-reservoir prevalence data were not available in the Indian literature; the corresponding sample sizes for these two diseases should be interpreted with this distinction in mind. Fourth, publication bias cannot entirely be excluded, particularly for diseases represented by a small number of studies, and may have led to over- or under-estimation of true prevalence for some pathogens. Nevertheless, this is the first comprehensive meta-analysis-guided sampling framework developed under a single platform for multiple priority diseases in India, providing an evidence-based foundation for future national surveillance programmes.

## Data Availability

Publicly available datasets were analysed in this study. This data can be found at: the data are from the literature available in the public domain.
