# Supplementary material for: A meta-analysis-guided sampling plan for estimating the prevalence of 10 zoonotic and 4 transboundary animal diseases across India
Source: Front Vet Sci. 2026 Oct 16;13:1919244. doi: 10.3389/fvets.2026.1919244 (PMC13720287; doi:10.3389/fvets.2026.1919244)
Supplement: Supplementary file 1 [file Table_1.docx]

**Supplementary Table S1. Full search strings used for the systematic literature search, by disease.**

| **No.** | **Disease** | **Full search string** |
| --- | --- | --- |
| 1 | **Brucellosis** | ("brucellosis" OR "Brucella" OR "Brucella abortus" OR "Brucella melitensis" OR "Brucella suis") AND ("prevalence" OR "seroprevalence" OR "incidence" OR "occurrence" OR "epidemiology" OR "infection") AND ("India" OR "Indian") |
| 2 | **Coxiellosis/Q-fever** | ("Q fever" OR "Q-fever" OR coxiellosis OR "Coxiella burnetii" OR "Coxiella burnetti") AND ("prevalence" OR "seroprevalence" OR "incidence" OR "occurrence" OR "epidemiology" OR "infection") AND ("India" OR "Indian") |
| 3 | **Cysticercosis** | ("cysticercosis" OR "Taenia solium" OR "Cysticercus cellulosae" OR "porcine cysticercosis") AND ("prevalence" OR "seroprevalence" OR "incidence" OR "occurrence" OR "epidemiology" OR "infection") AND ("India" OR "Indian") |
| 4 | **Crimean-Congo haemorrhagic fever (CCHF)** | ("Crimean-Congo haemorrhagic fever" OR "Crimean-Congo hemorrhagic fever" OR CCHF OR "Crimean Congo" OR "Congo fever") AND ("prevalence" OR "seroprevalence" OR "incidence" OR "occurrence" OR "epidemiology" OR "infection") AND ("India" OR "Indian") |
| 5 | **Scrub typhus** | ("scrub typhus" OR "scrub typhus fever" OR "Orientia tsutsugamushi" OR "Orientia tsutsugamushi infection" OR "tsutsugamushi disease") AND ("prevalence" OR "seroprevalence" OR "incidence" OR "occurrence" OR "epidemiology" OR "infection") AND ("India" OR "Indian") |
| 6 | **Swine influenza** | ("swine influenza" OR "swine flu" OR "swine influenza virus" OR "influenza A" OR "influenza A virus" OR "H1N1" OR "H3N2") AND ("prevalence" OR "seroprevalence" OR "incidence" OR "occurrence" OR "epidemiology" OR "infection") AND ("India" OR "Indian") AND ("pig" OR "pigs" OR "swine" OR "porcine") |
| 7 | **Tuberculosis** | ("tuberculosis" OR "Mycobacterium tuberculosis" OR "Mycobacterium bovis" OR "bovine tuberculosis" OR "animal tuberculosis") AND ("prevalence" OR "seroprevalence" OR "incidence" OR "occurrence" OR "epidemiology" OR "infection") AND ("India" OR "Indian") AND ("cattle" OR "buffalo" OR "livestock" OR "animal" OR "bovine") |
| 8 | **Japanese encephalitis** | ("Japanese encephalitis" OR "Japanese encephalitis virus" OR JEV OR "flavivirus" OR "Japanese B encephalitis") AND ("prevalence" OR "seroprevalence" OR "incidence" OR "occurrence" OR "epidemiology" OR "infection") AND ("India" OR "Indian") |
| 9 | **Listeriosis** | ("listeriosis" OR "Listeria monocytogenes" OR "Listeria infection" OR "Listeria") AND ("prevalence" OR "occurrence" OR "incidence" OR "contamination" OR "epidemiology" OR "infection") AND ("India" OR "Indian") |
| 10 | **Cryptosporidiosis** | ("cryptosporidiosis" OR "Cryptosporidium" OR "Cryptosporidium parvum" OR "Cryptosporidium infection") AND ("prevalence" OR "seroprevalence" OR "incidence" OR "occurrence" OR "epidemiology" OR "infection") AND ("India" OR "Indian") AND ("cattle" OR "buffalo" OR "calf" OR "calves" OR "livestock" OR "animal") |
| 11 | **Salmonellosis** | ("salmonellosis" OR "Salmonella" OR "Salmonella enterica" OR "Salmonella infection") AND ("prevalence" OR "seroprevalence" OR "incidence" OR "occurrence" OR "epidemiology" OR "infection") AND ("India" OR "Indian") |
| 12 | **Porcine reproductive and respiratory syndrome (PRRS)** | ("porcine reproductive and respiratory syndrome" OR "PRRS" OR "PRRSV" OR "porcine reproductive respiratory syndrome virus") AND ("prevalence" OR "seroprevalence" OR "incidence" OR "occurrence" OR "epidemiology" OR "infection") AND ("India" OR "Indian") AND ("pig" OR "pigs" OR "swine" OR "porcine") |
| 13 | **Lumpy skin disease (LSD)** | ("lumpy skin disease" OR "LSD" OR "lumpy skin disease virus" OR "LSDV" OR "capripoxvirus" OR "Capripox virus") AND ("prevalence" OR "seroprevalence" OR "incidence" OR "occurrence" OR "epidemiology" OR "infection") AND ("India" OR "Indian") AND ("cattle" OR "bovine" OR "livestock") |
| 14 | **African swine fever (ASF)** | ("African swine fever" OR "African swine fever virus" OR ASF OR ASFV) AND ("prevalence" OR "seroprevalence" OR "incidence" OR "occurrence" OR "epidemiology" OR "infection" OR "outbreak") AND ("India" OR "Indian") AND ("pig" OR "pigs" OR "swine" OR "porcine") |
