# Supplementary material for: A meta-analysis-guided sampling plan for estimating the prevalence of 10 zoonotic and 4 transboundary animal diseases across India
Source: Front Vet Sci. 2026 Oct 16;13:1919244. doi: 10.3389/fvets.2026.1919244 (PMC13720287; doi:10.3389/fvets.2026.1919244)
Supplement: Supplementary file 2 [file Table_2.docx]

**Supplementary Table S2. Diagnostic parameters used for each disease.**

| **Disease** | **Targeted test** | **Sensitivity (%)** | **Specificity (%)** | **Confidence interval (%)** |
| --- | --- | --- | --- | --- |
| Brucellosis | Competitive or blocking Ab ELISA | 97.9 | 97.24 | 95 |
| Coxiellosis/Q-fever | Indirect Ab ELISA | 96.2 | 94.47 | 95 |
| Cysticercosis | Sandwich Ag ELISA | 100 | 99.6 | 95 |
| CCHF | Indirect Ab ELISA | 99 | 100 | 95 |
| Scrub typhus | PCR | 90 | 90 | 95 |
| Swine influenza | Indirect Ab ELISA | 90 | 90 | 95 |
| Tuberculosis | Single intradermal test | 94.52 | 92.5 | 95 |
| Japanese encephalitis | Indirect Ab ELISA | 100 | 100 | 95 |
| Listeriosis | Isolation and PCR | 90 | 90 | 95 |
| Cryptosporidiosis | Sandwich antigen ELISA | 90 | 90 | 95 |
| Salmonellosis | Isolation and PCR | 99 | 90 | 95 |
| PRRS | Indirect Ab ELISA | 96 | 92.5 | 95 |
| LSD | Double-antigen sandwich ELISA | 90 | 90 | 95 |
| ASF | Indirect Ab ELISA | 90 | 94 | 95 |

**Note:** Sensitivity and specificity values were used based on target diagnostic performance criteria through expert consensus, prioritizing WOAH-recommended and/or internationally validated, commercially marketed assays, rather than a single published source (performance varies by manufacturer, kit version, and study population).
